# Salivary Immune Responses to the 7-Valent Pneumococcal Conjugate Vaccine in the First 2 Years of Life

**DOI:** 10.1371/journal.pone.0046916

**Published:** 2012-10-16

**Authors:** Gerwin D. Rodenburg, Elisabeth A. M. Sanders, Elske J. M. van Gils, Reinier H. Veenhoven, Tomasz Zborowski, Germie P. J. M. van den Dobbelsteen, Andries C. Bloem, Guy A. M. Berbers, Debby Bogaert

**Affiliations:** 1 Department of Pediatric Immunology and Infectious Diseases, Wilhelmina Children's Hospital, University Medical Center Utrecht, Utrecht, The Netherlands; 2 Linneaus Institute, Spaarne Hospital, Hoofddorp, The Netherlands; 3 National Institute for Public Health and the Environment, Bilthoven, The Netherlands; 4 Department of Immunology, University Medical Center Utrecht, Utrecht, The Netherlands; Rockefeller University, United States of America

## Abstract

**Background:**

The CRM197-conjugated 7-valent pneumococcal vaccine (PCV7) is protective against vaccine serotype disease and nasopharyngeal carriage. Data on PCV7-induced mucosal antibodies in relation to systemic or natural anticapsular antibodies are scarce.

**Methods:**

In a randomized controlled setting, children received PCV7 at age 2 and 4 months (2-dose group), at age 2, 4 and 11 months (2+1-dose group) or no PCV7 (control group). From 188 children paired saliva samples were collected at 12 and 24 months of age. From a subgroup of 15 immunized children also serum samples were collected. IgG and IgA antibody-levels were measured by multiplex immunoassay.

**Results:**

At 12 months, both vaccine groups showed higher serum and saliva IgG-levels against vaccine serotypes compared with controls which sustained until 24 months for most serotypes. Salivary IgG-levels were 10–20-fold lower compared to serum IgG, however, serum and saliva IgG-levels were highly correlated. Serum and salivary IgA-levels were higher in both vaccine groups at 12 months compared with controls, except for serotype 19F. Higher salivary IgA levels remained present for most serotypes in the 2+1-dose group until 24 months, but not in the 2-dose group. Salivary IgA more than IgG, increased after documented carriage of serotypes 6B, 19F and 23F In contrast to IgG, salivary IgA-levels were comparable with serum, suggesting local IgA-production.

**Conclusions:**

PCV7 vaccination results in significant increases in salivary IgG and IgA-levels, which are more pronounced for IgG when compared to controls. In contrast, salivary anticapsular IgA-levels seemed to respond more to natural boosting. Salivary IgG and IgA-levels correlate well with systemic antibodies, suggesting saliva might be useful as potential future surveillance tool.

## Introduction

Protein-conjugated pneumococcal vaccines (PCVs) are effective against vaccine serotype invasive pneumococcal disease (IPD), as well as pneumonia and acute otitis media (AOM) [1]–[3]. Besides protection against disease, systemic administration of PCV results in a reduction of nasopharyngeal vaccine serotype pneumococcal acquisition and colonization [4], [5]. Vaccine-induced systemic anticapsular IgG antibodies, which activate complement and enhance phagocytosis, are presumed to mediate protection against IPD [6]. For nasopharyngeal colonization systemic serotype-specific IgG levels are reported to be inversely related to new nasopharyngeal acquisition of the given serotype [7], [8]. Serological IgG levels as ‘correlates of protection’ against AOM and carriage have been suggested although they are not well defined yet [9]–[11].

At the mucosal surface, anti-capsular IgA antibodies have been shown to support complement-dependent opsonophagocytosis, and agglutination of the pneumococcus [12], [13]. IgA antibodies against pneumococcal surface proteins also have been described as major contributor in protection against mucosal disease [14]. The role of anticapsular mucosal antibodies after systemic PCV immunization in protection against pneumococcal disease and carriage is however less clear. Besides systemic IgG, PCVs also induce IgG and IgA antibody in saliva, reflecting efficacy at the mucosal level. The magnitude and dynamics of these salivary antibodies however are largely unknown [15]–[19]. Most studies on salivary antibodies lack unvaccinated control groups and since salivary antibody responses are also enhanced by natural pneumococcal carriage this hampers full estimation of vaccine impact [11], [13], [14]. Furthermore, studies were often restricted to few serotypes [15], [16] with limited data on persistence and boostability of salivary antibody levels [18], [19]. Finally, in most published studies salivary antibody levels were difficult to measure, possibly due to the used EIA or ELISA detection-method. This restricted study observations and allowed for the description of rough vaccine effects only [15], [16], [19]

In this study, we applied a fluorescent bead-based multiplex immuno assay (MIA) using LUMINEX technology [20] to determine salivary IgG and IgA anticapsular antibody levels. Responses against 11 vaccine and non-vaccine serotypes were measured in a large group of children participating in a randomized controlled trial on reduced-dose schedules with the 7-valent CRM197-conjugated pneumococcal vaccine (PCV7) [4]. Paired salivary samples were collected at the age of 12 and 24 months from vaccinees and unvaccinated controls. Also,we studied the effect of natural exposure to pneumococcal carriage on homologous mucosal IgG and IgA levels in the unvaccinated children. Finally, in a small subgroup we studied the association between serum and saliva anticapsular antibody levels.

## Methods

### Ethics Statement

The study was approved by a national medical ethics committee (Stichting Therapeutische Evaluatie Geneesmiddelen, http://www.stegmetc.org) and undertaken in accordance with the European Statements for Good Clinical Practice, which includes the provisions of the Declaration of Helsinki of 1989.

### Study design

Between July 2005 and February 2006, before nationwide implementation of PCV7 in the National Immunization Program (June 2006) in the Netherlands, 1005 infants were enrolled in a randomized controlled trial investigating the effects of reduced-dose PCV7 schedules on pneumococcal carriage during the first two years of life (NCT00189020) [4]. Healthy infants younger than 12 weeks of age, not yet having received any infant vaccination were eligible for inclusion. Groups of infants received the following vaccination schedules, (a) two primary doses of PCV7 at 2 and 4 months of age (2-dose group); (b) two primary doses at 2 and 4 months followed by a booster dose at 11 months of age (2+1-dose group); (c) no PCV7 vaccination (control group). Following randomization, study participants were asked to voluntary participate in a saliva sub-study. The first sixty participants per study group that gave permission to collect saliva were enrolled, and samples were collected at both 12 and 24 months of age using oral swabs (Malvern, Worchester UK). Salivary fluid was immediately squeezed from the swab and immediately frozen by snap-freezing on dry ice (carbon dioxide −78°C). At the study site the salivary fluid was stored at −80°C. From a separate subgroup of children permission was asked to obtain a serum sample either at 12 and/or at 24 months of age for the purpose of studying serum antibody responses (published previously [21]). Serum was separated within 24 hours and stored at −20°C until assayed. Eventually, we had paired saliva- and blood-samples only from a small set of immunized participants, i.e. 15 children. From all children in the study, nasopharyngeal swabs were obtained consecutively at the age of 6 weeks and at 6, 12, 18 and 24 months. Overall carriage results were described earlier [4]. Identification of *S pneumoniae* nasopharyngeal carriage was based on colony morphology and conventional methods of determination [4]. Written informed consent was obtained from the parents or guardians of all study participants. Laboratory personnel were unaware of treatment allocation, and the randomization key was not disclosed until the study was completed.

### Study Vaccines

The licensed 7-valent CRM197-conjugated pneumococcal vaccine (Prevenar™ Pfizer/Wyeth), containing pneumococcal polysaccharides 4, 6B, 9V, 14, 18C, 19F and 23F, was administered during regular well baby-clinic visits, together with routine DTaP-IPV-Hib immunizations according to the Dutch NIP [22].

### Multiplex immunoassay

IgG and IgA antibody levels in serum and saliva were measured by a multiplex fluorescent bead-based immunoassay (MIA) using LUMINEX technology, for which the protocol and validation data were described previously [20]. In short, eleven sets of microspheres were coated with the pneumococcal polysaccharide antigens 4, 6B, 9V, 14, 18C, 19F and 23F (serotypes covered by PCV7) and 1, 3, 5, 7F (non-vaccine serotypes) (ATCC; Manassas, VA). Antigens were conjugated to Poly-L-lysine, after which the complex was attached to the microspheres by a reaction using EDC with sulpho-NHS. Standard reference serum (89SF-5; FDA) with known antibody concentrations for 23 pneumococcal capsular polysaccharides was used for standard serial dilutions in duplo. Salivary samples were thawed and centrifuged. Supernatants were diluted 1∶1 using 5% antibody-depleted human serum containing cell wall polysaccharide and 22F polysaccharide (ADHS-CWPS Multi; Statens Serum Institut), and incubated at 4°C. Samples were tested in duplo with a minimum of 2 blank wells per run. Sera samples were diluted 1∶100 and 1∶1000 using 5% ADHS-CWPS Multi and incubated at 4°C with shaking. From each diluted sample 2 times 25 µl was mixed with an equal volume of beads. Goat-anti-human-IgG-PE or goat-anti-human-IgA-PE solution 1∶200 (Jackson Immuno Research) was added. After a final wash, analysis of the beads was performed on a BioPlex 100 apparatus (Bio-Rad) using the BioPlex software package (version 4.1.1; Bio-Rad). Antibody levels were expressed in ng/ml IgA or IgG. The cut-off for positivity (2 SD of 20 blank wells) varied for IgG antibodies between 0.15 ng/ml (serotype 7F) and 1.35 ng/ml (serotype 9V) and for IgA antibodies between 0.03 ng/ml (serotype 23F) and 0.49 ng/ml (serotype 3). Samples below the cutt-off were assigned half the detection limit for the given serotype. In addition, total protein concentration in saliva was determined by the Pierce BCA Protein Assay Kit (Rockford) and salivary antibody concentrations were calculated per µg total protein.

### Statistical Analysis

Salivary IgG and IgA antibody levels are expressed in geometric mean concentrations (GMC; ng/ml) with 95% CI. Statistical differences between IgG and IgA GMC values were assessed by log transformed unpaired t test. Antibody concentrations with paired saliva samples taken at 12 and 24 months were compared by log transformed paired t test. Correlations between salivary and serum antibody levels were assessed by Spearman correlation. For analyses of potential correlations between previous carriage and consecutive antibody responses in unvaccinated controls, we focused on the 3 most frequently carried serotypes: 6B, 19F and 23F. Children were defined as colonized when one or more positive cultures were obtained for that serotype up to the moment of saliva sampling (i.e. 12 or 24 months of age). The children with serotype-negative nasopharyngeal samples up to the moment of saliva sampling were defined as non-colonized. All reported *p*-values are 2-sided, *p*-values smaller than 0.05 were considered significant. Analyses were performed with SPSS 15.0.

## Results

### Study participants

Of the 1005 enrolled children in the main carriage trial, 187 children participated in the saliva immunogenicity study. Because of limited volumes of saliva obtained of some individuals, salivary IgG antibodies were measured in 177 and 175 samples and salivary IgA antibodies in 166 and 158 samples at 12 and 24 months, respectively (see Table 1 for baseline characteristics and sample numbers per group). No major differences in demographic characteristics and risk factors for pneumococcal carriage were found between groups of children participating in the main trial and the saliva [4]. We analysed natural and vaccine-induced salivary anticapsular antibodies as absolute quantity (ng/ml), and corrected for dilution factor by normalizing for total protein concentration in saliva (ug/ml). Correction for salivary total protein resulted in similar observations and conclusions compared to results based on absolute quantity of salivary antibody levels (data not shown). We therefore further present data using plain antibody concentrations for our analyses.

**Table 1:** Baseline characteristics of participants receiving 2 doses, 2+1-doses or no PCV7 vaccinations (controls).

	Study group		
	Controls	2-dose	2+1-dose
	n = 61	n = 61	n = 65
Male (%)	29 (48)	26 (43)	37 (57)
Age at vaccination; mean (SD), mo			
PCV 2 mo	-	2.1 (0.2)	2.1 (0.2)
PCV 4 mo	-	4.3 (0.4)	4.2 (0.3)
PCV 11 mo	-	-	11.1 (0.3)
Age at saliva sampling; mean (SD), mo			
12 mo	11.9 (0.3)	12.0 (0.3)	12.1 (0.3)
24 mo	24.5 (0.8)	24.4 (0.6)	24.2 (0.5)
Sample taken (%)			
12 mo	60 (98)	54 (89)	63 (97)
24 mo	55 (90)	58 (95)	62 (95)
Siblings present (%)			
12 mo	28 (46)	36 (59)	36 (55)
24 mo	35 (58)	40 (66)	40 (64)
Daycare attendance* (%)			
12 mo	40 (66)	34 (56)	31 (48)
24 mo	44 (72)	37 (61)	40 (62)
Passive tobacco smoke exposure (%)			
12 mo	3 (5%)	5 (8%)	3 (5%)
24 mo	2 (3%)	5 (8%)	5 (8%)

* Defined as more than 4 hours per week with at least 1 child from a different family.

### Salivary vaccine serotype IgG antibody levels

At the age of 12 months of age, salivary IgG GMC antibody values varied between 1.1 ng/ml (serotype 18C) and 11.2 ng/ml (serotype 19F) in unvaccinated control children (Table 2). Significantly higher IgG GMC values were found for all 7 vaccine serotypes in the 2-dose group compared with the control group (p<0.001). A further increase in IgG GMC values was observed in the 2+1-dose group 1 month after the booster PCV7 at 11 months compared with the 2-dose group for all serotypes (p<0.001).

**Table 2:** Salivary IgG antibody levels (GMC; ng/ml) in children after 2 doses, 2+1-doses of PCV7 or no PCV7 vaccinations (controls) at the age of 12 and 24 months.

	12 months				24 months			
	Controls	2-dose	2+1-dose		Controls	2-dose	2+1-dose	
Serotype	n = 60	n = 54	n = 63	p-Values	n = 55	n = 58	n = 62	p-Values
**4**	1.8	11.3	124.0	**α, β, γ**	1.5	6.0	14.8	**α, β, γ**
**6B**	4.4	12.7	188.2	**α, β, γ**	7.1	40.4	67.0	**β, γ**
**9V**	2.6	18.7	177.9	**α, β, γ**	2.7	11.8	34.3	**α, β, γ**
**14**	3.1	49.7	360.3	**α, β, γ**	3.2	15.7	52.3	**α, β, γ**
**18C**	1.1	11.5	137.3	**α, β, γ**	2.0	8.8	19.0	**α, β, γ**
**19F**	11.2	33.8	147.2	**α, β, γ**	19.7	72.3	85.7	**β, γ**
**23F**	1.2	8.2	112.4	**α, β, γ**	2.0	12.8	39.7	**α, β, γ**
**1**	4.6	3.0	3.7		4.3	4.1	5.7	
**3**	1.2	1.3	0.7		1.0	1.4	1.5	
**5**	8.6	7.3	9.1		11.2	10.7	12.9	
**7F**	5.1	3.3	3.5		4.2	3.1	5.3	

α *p*-Values<0.05; 2+1 vs. 2-dose schedule.

β *p*-Values<0.05; 2+1-dose vs. controls.

γ *p*-Values<0.05; 2-dose vs. controls.

Calculated using log transformed unpaired t test, p-values are 2 sided.

**Figure 1:**
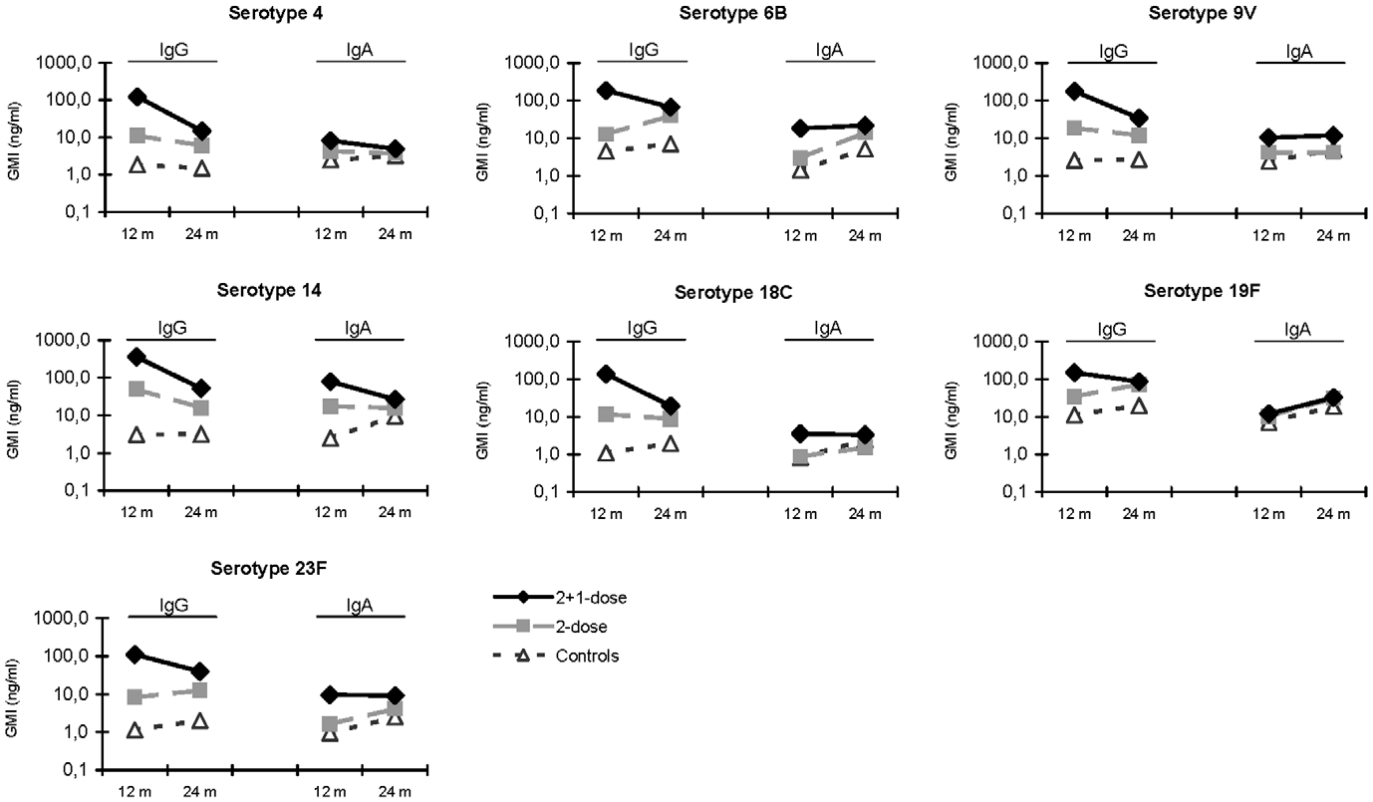
Salivary IgG and IgA antibody levels (GMC; ng/ml) against vaccine serotypes in children after 2 doses, 2+1-doses of PCV7 or no PCV7 vaccinations (controls) at the age of 12 and 24 months.

In 24 months-old controls, natural salivary IgG GMC values had increased for the serotypes 6B, 18C, 19F and 23F, where GMC values of the remaining serotypes were comparable to 12-month-old values (Figure 1). In immunized children (2-dose group) at 24 months of age, IgG GMC values also had increased for serotypes 6B, 19F and 23F but decreased for vaccine serotypes 4, 9V and 14 compared with values at 12 months of age (Figure 1). Still up to 24 months of age, salivary GMC values against all vaccine serotypes were higher in the 2-dose group compared to controls. In the 2+1-dose group, IgG GMC values had declined for all vaccine serotypes at 24 months of age (p<0.05), although GMC values remained significantly higher compared to the 2-dose group, except for serotypes 6B and 19F.

### Salivary vaccine serotype IgA antibody levels

At the age of 12 months, salivary IgA GMC antibody values varied between 0.8 ng/ml (serotype 18C) and 7.2 ng/ml (serotype 19F) in unvaccinated control children (Table 3). Compared to the control group, significantly higher IgA GMC values were found in saliva of the 2-dose group for 4 of the 7 vaccine serotypes. However, no differences were observed for serotype 18C, 19F and 23F. In the 2+1-dose group salivary IgA GMC values were also higher for 6 of the 7 vaccine serotypes compared to the 2-dose group at 12 months of age. For serotype 19F salivary IgA levels were high in all study groups but did not significantly differ between the 2+1-dose group, 2-dose and control group.

**Table 3:** Salivary IgA antibody levels (GMC; ng/ml) in children after 2 doses, 2+1-doses of PCV7 or no PCV7 vaccinations (controls) at the age of 12 and 24 months.

	12 months				24 months			
	Controls	2-dose	2+1-dose		Controls	2-dose	2+1-dose	
Serotype	n = 57	n = 51	n = 58	p-Values	n = 49	n = 50	n = 59	p-Values
**4**	2.5	4.3	8.2	**α, β, γ**	3.2	3.8	4.9	β
**6B**	1.4	2.9	18.4	**α, β, γ**	5.1	14.2	22.0	β, γ
**9V**	2.4	4.1	10.3	**α, β, γ**	4.9	4.2	11.8	α, β
**14**	2.5	17.5	78.3	**α, β, γ**	9.5	15.6	26.6	β
**18C**	0.8	0.9	3.6	**α, β**	2.4	1.5	3.3	α
**19F**	7.2	10.0	11.8		18.6	31.0	32.0	
**23F**	1.0	1.6	9.6	**α, β**	2.5	4.2	9.2	β
**1**	4.4	3.9	3.1		4.8	4.0	4.2	
**3**	8.6	12.0	8.3		9.7	11.9	14.9	
**5**	4.8	4.1	4.3		6.2	5.2	6.8	
**7F**	4.7	3.7	3.4		6.4	4.8	6.3	

α *p*-Values<0.05; 2+1 vs. 2-dose schedule.

β *p*-Values<0.05; 2+1-dose vs. controls.

γ *p*-Values<0.05; 2-dose vs. controls.

Calculated using log transformed unpaired t test, p-values are 2 sided.

At the age of 24 months natural salivary IgA GMC values against all vaccine serotypes had increased iIn the control group when compared with 12 months of age (Figure 1). In the 2-dose group IgA values in saliva had increased for the serotypes 6B, 19F and 23F at 24 months of age, though. No significant differences in IgA values were observed between the 2-dose group and controls, except for serotype 6B (Table 3). In the 2+1-dose group IgA GMC values for serotypes 4 and 14 declined at 24 months of age compared with 12 months, while serotypes 6B, 9V, 18C and 23F remained at the same level. Overall, this resulted in higher IgA values against 5/7 vaccine serotypes in saliva of the 2+1-dose group compared with controls, except for serotypes 18C and 19F. Compared to the 2-dose group, 2+1-doses showed significantly higher IgA salivary antibodies for the serotypes 9V and 18C. Like at 12 months of age, serotype 19F showed similar salivary IgA GMC values in all 3 randomization groups at the age of 24 months (Table 3).

### Salivary non-vaccine serotype antibody levels

At the age of 12 months salivary IgG GMC values against non-vaccine serotypes did not differ between the different randomization groups (Table 2). At the age of 24 months, IgG GMC values for the non-vaccine serotypes increased in the 2-dose group for serotype 5 and in the 2+1-dose group for all 4 non-vaccine serotypes compared with values at 12 months. However, these increases did not result in significant differences between the 3 randomization groups (Table 2).

IgA GMC values against non-vaccine serotypes at the age of 12 months did not differ between the 3 randomization groups (Table 3). Salivary IgA GMC values increased in the control group at 24 months for serotypes 5 and 7F and in the 2+1-dose group for the serotypes 3, 5 and 7F. Like salivary IgG, no significant differences in salivary IgA GMC values were observed between the 3 randomization groups at 24 months (Table 3).

### Development of salivary antibodies in relation to carriage in unvaccinated controls

**Figure 2:**
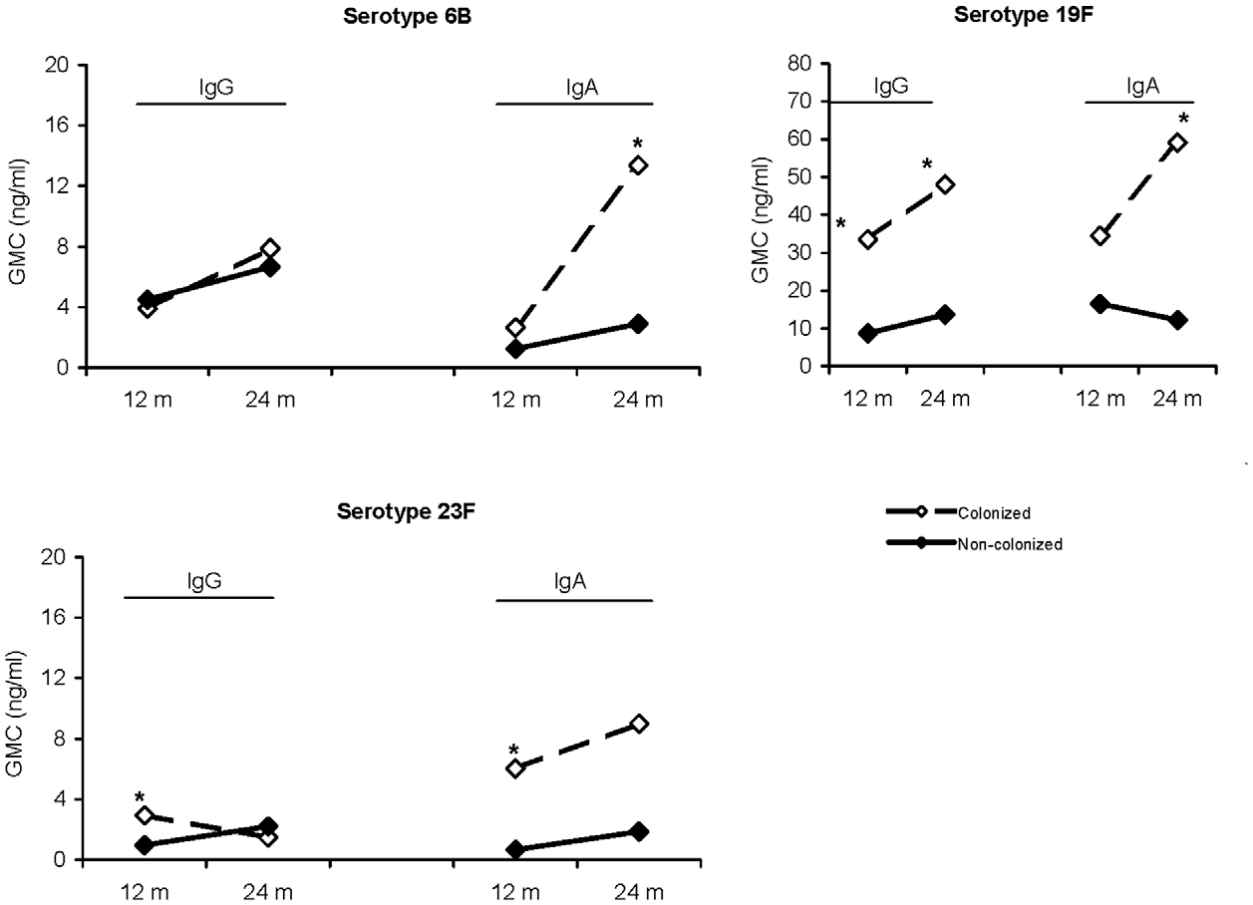
Salivary IgG and IgA levels (GMC; ng/ml) at 12 or 24 months of age in unvaccinated children after previously being colonized with the homologous serotype at 6 weeks, 6, 12, 18 or 24 months of age. Closed squares: serotype-negative children, open squares: children with serotype-positive swabs. *Significant difference; p<0.05.

We analysed nasopharyngeal pneumococcal carriage at the age of 6 weeks and 6, 12, 18 and 24 months. In the PCV7 unvaccinated control group salivary antibody levels and carriage data were available for 60 and 55 children at 12 and 24 months, respectively. Nasopharyngeal carriage at the age of 6 weeks, 6 months or 12 months was found most frequently for serotypes 6B, 19F and 23F; 9 of 60 children (15%) had been positive for serotype 6B, 11 children (18%) for serotype 19F and 9 children (15%) for serotype 23F. At 12 months of age, higher homologous salivary IgG and IgA GMC values were found in previously colonized children for serotypes 19F and 23F but not for serotype 6B when compared to salivary IgG and IgA GMCs in children with no culture-proven carriage of the respective serotypes (Figure 2).

After additional cultures were taken at 18 and 24 months, the proportion of carriers in the unvaccinated control children had risen to 21 (38%) for serotype 6B, 16 children (29%) for serotype 19F and 11 children (20%) for serotype 23F. Higher homologous salivary IgG GMC values were observed for serotype 19F in carriers only at 24 months compared to children with 19F serotype-negative swabs (Figure 2). With respect to salivary IgA, significantly higher homologous levels were found in previously colonized children with serotypes 6B or 19F,with borderline significance for serotype 23F (p = 0.078).

Lastly, we analysed the association of salivary IgA and IgG antibody levels at 12 months and protection against nasopharyngeal pneumococcal carriage at the age of 18 and 24 months. In our data no such association could be proven for serotype 6B, 19F and 23F (data not shown).

### Serum vs. salivary antibodies

From a small group of 15 immunized children simultaneous saliva and serum samples were available. A positive correlation was found between serum and salivary IgG antibody levels for all vaccine serotypes (range *r* = 0.54 for serotype 14 to *r* = 0.88 for serotype 6B) (Table 4). Also positive correlations between serum and salivary IgA antibody levels were observed (range *r* = 0.57 for serotype 4 to *r* = 0.92 for serotype 6B). For the non-vaccine serotypes no positive correlation between serum and saliva IgG or IgA antibody levels existed. For vaccine serotypes 11.0 to 26.2-fold higher IgG levels were observed in serum compared to saliva (Table 5). In contrast, for IgA the highest serum/saliva-ratio was 2.0 (serotype 23F). For serotype 19F even higher salivary IgA levels were observed compared to serum levels (serum/saliva-ratio 0.4).

**Table 4:** Relation between serum and salivary IgG and IgA antibody levels in the PCV7 vaccinated groups.

	IgG Serum-Saliva		IgA Serum-Saliva	
	(n = 15)		(n = 15)	
Serotype	r	P	r	p
4	0.63	**0.011**	0.57	**0.032**
6B	0.88	**<0.001**	0.92	**<0.001**
9V	0.64	**0.010**	0.71	**0.005**
14	0.55	**0.035**	0.66	**0.010**
18C	0.79	**<0.001**	0.65	**0.012**
19F	0.57	**0.027**	0.77	**0.001**
23F	0.87	**<0.001**	0.69	**0.007**
1	0.32	0.248	0.40	0.160
3	−0.06	0.824	0.26	0.375
5	0.38	0.164	0.20	0.483
7F	0.31	0.254	0.30	0.296

Correlations between salivary and serum antibody levels were assessed by Spearman correlation. All reported *p*-values are 2-sided, *p*-values smaller than 0.05 were considered significant and are depicted in bold.

**Table 5:** Serum/saliva ratio for serotype-specific IgG and IgA antibodies in the PCV7 vaccinated groups.

	Serum/Saliva ratio*	
Serotype	IgG	IgA
4	26.2	1.8
6B	11.0	1.4
9V	17.0	1.1
14	22.7	0.9
18C	18.8	1.8
19F	11.6	0.4
23F	24.5	2.0
1	27.8	1.7
3	141.7	1.4
5	5.8	0.6
7F	15.9	0.8

* Serum/saliva ratio's presented in geomeans.

## Discussion

We studied salivary IgG and IgA antibody responses in PCV7 vaccinated and non-vaccinated children in a randomized controlled setting before nationwide PCV7 introduction in the Netherlands. We also studied the nasopharyngeal carriage of these children, which allowed us to study the impact of serotype-specific carriage on salivary antibody responses (for detailed carriage data see [4]). We showed that in unvaccinated controls salivary anticapsular IgG and IgA antibodies were induced by natural boosting via carriage for most serotypes. Earlier it was already observed by Simell et al that natural exposure to pneumococci induced salivary IgA antibody responses [23]. Our study confirms this observation, plus shows this is also true for boosting of salivary IgG. Until now, this association between salivary IgG antibodies and carriage was not observed, possibly because of the low salivary IgG levels which are commonly below the lower limit of detection when methods like EIA are used.

Systemic administration of 2 primary doses of PCV7 resulted in increased salivary IgG antibody responses against all vaccine serotypes compared to unvaccinated controls. An additional PCV7 booster dose at 11 months increased salivary IgG antibody levels one month later compared to 2 primary doses, illustrating that pneumococcal conjugate vaccines do contribute to salivary IgG antibody levels [18]. For salivary IgA different dynamics in salivary antibody vaccine responses were observed. At 24 months of age the parallel increase in serotype-specific salivary IgA levels in the 2-dose and unvaccinated control group resulted in less pronounced differences between randomization groups, suggesting a parallel process of natural boosting. Our data even show that carriage-induced salivary IgA levels seems comparable to vaccine-induced IgA levels, which was most clear for serotype 19F. After PCV vaccinations, less vaccine serotype-specific carriage occurs [4]. This might lead to less natural boosting of IgA anticapsular antibody levels compared to the control group, and can explain why also most previous studies do not observe a difference in salivary IgA levels between PCV7 vaccinated and unvaccinated children [16]–[18].

No significant differences were observed in non-vaccine serotype salivary antibody responses between controls and vaccinees, which is in correspondence with earlier reports [18]. Interestingly, the increases in antibody levels against non-vaccine serotypes between 12 and 24 months of age, especially in the 2+1-dose group, most probably represent natural boosting. Although there is potentially more carriage of non-vaccine serotypes in the vaccinated groups, none of these tested serotypes were frequently encountered in conventional cultures in our carriage study [4]. This might be due to large intervals between samples, however, polyreactivity on the same antigenic stimulus can not be ruled out [8], as well as cross-reactivity with other bacteria [24], [25].

The exact contribution of PCV7 induced salivary IgA and IgG antibody levels in protection against nasopharyngeal carriage and disease is not yet known. We reported a 58% and 60% reduction in vaccine serotype pneumococcal carriage at the age of 24 months in both the 2-dose and 2+1-dose group compared to unvaccinated controls, respectively [4]. In the present study however at this age no difference in salivary IgA antibody levels between the 2-dose and controls could be observed for most vaccine serotypes. This in contrast to significantly higher salivary IgG antibodies antibodies observed in vaccinated children where, in addition, a stepwise increase was observed between the control, 2-dose and 2+1-dose groups. This may suggest that vaccine-induced anti-capsular salivary IgG antibodies have a stronger contribution to protection against pneumococcal colonization then IgA. However, one has to realize that level of antibodies may not represent functionality [3] and IgA antibodies have been shown to support anti-capsular complement-dependent opsonophagocytosis, and agglutination of the pneumococcus at the mucosal surface; functions which are not tested with our quantitative assay [12], [13].

Lastly, we studied the potential correlation between serum and saliva antibodies in a small subgroup of children of which both saliva- and serum-samples were available. Although the small sample size does not allow for firm conclusions, we observed that after PCV7 administration salivary IgG antibody levels correlated well with serum antibody levels, supporting the hypothesis of IgG transport to the mucosal site [15], [23]. In PCV7 vaccinated children systemic IgG levels proved to be 10–20 fold higher than salivary IgG levels, in contrast to the IgA levels. For IgA the serum/saliva-ratio's are suggestive for a predominance of local production of IgA at the mucosal site. Especially for serotype 19F where higher salivary IgA levels were observed compared with serum levels supports this hypothesis. Also we were able to show both IgG and IgA salivary antibody levels increased with serotype 19F carriage.

The fact that serum and salivary IgG correlate well after PCV7 vaccination immediately raises two new questions: the first. whether this method could potentially be used as surveillance strategy; collection of saliva is less invasive, easy to obtain in sufficient quantities, and methods for antibody analysis are similar for saliva and serum. It might therefore be a reasonable alternative whenever invasive measurements are impossible. Second, whether measurement of salivary IgG levels might elicit a cut-off for protection against carriage. However, new studies with a larger cohort of participants should be executed to get a better insight into the answers to both questions.

This study was performed well before herd effects after PCV7 introduction in the Dutch national immunization program. In the coming years the impact of decreased vaccine serotype carriage on salivary antibody responses have to be evaluated, as natural boosting seems important in salivary antibody persistence. Salivary vaccine responses may therefore vary between regions and continents. Also primary doses at older ages, more doses or broader intervals between doses can impact vaccine immunogenicity, as well as other factors like concomitant childhood vaccinations or ethnic background variability [21], [26]–[28]. Some limitations of this study should be addressed. First, salivary IgA is susceptible to cleavage through bacterial IgA1 proteases [29]. To prevent this, samples were immediately frozen after collection and analyzed directly after thawing. The randomized controlled setting also contributes to the reliability of the observed differences between groups. Second, due to the large 6-months interval of nasopharyngeal sampling, in between we will have missed carriage episodes in individuals. This might be the reason why not at all time points higher salivary antibody levels in colonized children could be observed compared with non-colonized children. Finally, the single colony method for serotyping may have resulted in missed multiple serotype carriage. Still for all of the 3 tested serotypes boosting of the immune system after natural exposure could be shown.

Strengths of our study include the randomized controlled study design with an unvaccinated control group. This made it possible to estimate the effect of PCV7 administration and pneumococcal carriage on salivary responses without the influences of temporal or geographical trends in distribution of circulating pneumococcal serotypes and to differentiate between true vaccine effects and natural immunity. Second, the highly sensitive multiplex technique (MIA) [30] allowed to obverse less robust differences between the different time points and groups. Lastly IgA antibody levels strongly depend on the secretion flow rate of the participant during sample collection [29], in this study antibody levels were also corrected for total salivary protein, which did not change the results.

In conclusion, systemic administration of PCV7 proved to induce both salivary IgG and IgA antibodies. IgG antibody levels remained higher after 2+1-doses up till 24 months of age, while for IgA a strong increase was observed with age, independent of immunization. Nasopharyngeal carriage proved to be a major contributor to salivary antibody levels, especially IgA. We would like to advocate for new studies on salivary antibodies as potential immunological correlates of protection against pneumococcal carriage as well as on the potential usefulness of salivary antibodies in vaccine monitoring to further support this work.,.
